# Monomers and Macromolecular Materials from Renewable Resources: State of the Art and Perspectives †

**DOI:** 10.3390/molecules27010159

**Published:** 2021-12-28

**Authors:** Alessandro Gandini, Talita M. Lacerda

**Affiliations:** 1 Graduate School of Engineering in Paper, Print Media and Biomaterials (Grenoble INP-Pagora), University Grenoble Alpes, LGP2, CEDEX 9, 38402 Saint Martin d’Hères, France; 2 Biotechnology Department, Lorena School of Engineering, University of São Paulo, Lorena CEP 12602-810, SP, Brazil; talitalacerda@usp.br

**Keywords:** polymers, biomass, polysaccharides, lignin, plant oils, furans, terpenes

## Abstract

A progressively increasing concern about the environmental impacts of the whole polymer industry has boosted the design of less aggressive technologies that allow for the maximum use of carbon atoms, and reduced dependence on the fossil platform. Progresses related to the former approach are mostly based on the concept of the circular economy, which aims at a thorough use of raw materials, from production to disposal. The latter, however, has been considered a priority nowadays, as short-term biological processes can efficiently provide a myriad of chemicals for the polymer industry. Polymers from renewable resources are widely established in research and technology facilities from all over the world, and a broader consolidation of such materials is expected in a near future. Herein, an up-to-date overview of the most recent and relevant contributions dedicated to the production of monomers and polymers from biomass is presented. We provide some basic issues related to the preparation of polymers from renewable resources to discuss ongoing strategies that can be used to achieve original polymers and systems thereof.

## 1. Introduction

Since the start of the large-scale production of synthetic polymers in the first half of the XX century, an exponential increase in their demand has taken place. Such a phenomenon can be easily explained by the low production costs and the excellent properties of polymers, which are often superior to any other material designed by man or available in nature. The global production of polymeric materials, which was approximately 1.5 million tons in 1950, reached 367 million tons in 2020 [1], to address the needs of construction, cars and airplanes, fabrics, electronics, packaging, and pharmaceutical industries, among many others [2]. However, their extensive use does not occur without consequences. While the incorrect disposal and increasing accumulation of synthetic polymers in the environment is a major threat to biodiversity and to human beings, their non-renewable origin implies the release of greenhouse gases, whose relationship with the increase of the average temperature of the planet has been clearly demonstrated [3,4].

In this context, the research and development of polymeric materials that have reduced environmental impacts, from the production to the disposal, have become very relevant [5,6,7]. Ideally, synthetic polymers should be designed with an increasing contribution of monomers produced from renewable resources, with minimum energy costs, minimum use of harmful reagents, and controlled release of harmful by-products. Moreover, the end-of-life stage must be kept on track, so that the polymer-based materials should be biodegradable or easily recyclable. It is, therefore, essential that the idealization, development, and production of polymers are aligned with the principles of green and sustainable chemistry.

**Figure 1:**
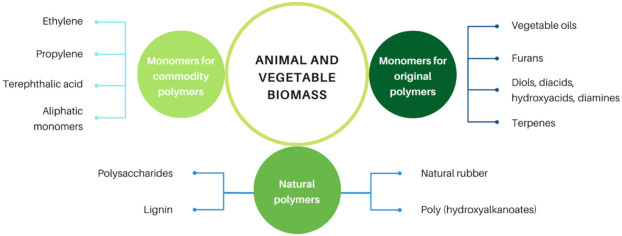
Three main approaches to produce monomers and macromolecules from renewable resources.

Herein, we aim to offer an overview of the field of *polymers based on renewable resources*, and its potential progress for the near future. Three major approaches are considered, as represented in Figure 1: (i) the exploitation of natural polymers to generate novel materials, (ii) the possibility of producing commodity polymers, which are traditionally prepared from fossil resources, using monomers derived from renewable counterparts, and (iii) the use of a variety of monomers and macromonomers derived from renewable resources to prepare original macromolecular structures with unique properties and potential applications in different industrial sectors. As this monograph is intended to give perspectives on the topic, the reader is encouraged to gather detailed information on the specific chemistry and other fundamental aspects in very rich contributions published by experts in the last decade [8,9,10,11,12,13,14,15,16,17,18,19,20,21,22,23].

## 2. It All Started with Natural Polymers

The exploitation of natural macromolecules in clothing, shelter, heating, and artifact manufacture, using both vegetable and animal resources, is as old as mankind itself, that took advantage of wood, resins, fibers, leather, silk, and other naturally available materials, to build primitive objects that contributed largely to the survival and development of the species. Moving forward to a somehow still empirical manipulation of matter, the XIX century was a transformative period, with important advances related to the vulcanization of natural rubber and to the chemical conversion of cellulose into processable materials such as nitrocellulose, cellulose acetates, and rayon, albeit without the actual recognition of their macromolecular structure, which was only irrefutably demonstrated in the early 1920s. Therefore, such polymers from renewable resources preceded and inspired the development of the first fossil-based counterparts [6].

Macromolecules are very abundant in nature, with examples that may vary from a strong association between cellulose, lignin, and hemicelluloses building the structure of plant cell wall, to RNA and DNA, which carry the genetic information of all living organisms. Such macromolecules differ enormously in terms of their chemical constitution, which allows a multitude of properties and natural functions. Then, exploiting the possibilities of their physical or chemical modifications is a smart approach to produce original materials. In the following paragraphs, a succinct outlook is presented to highlight the very broad potential provided by some naturally available macromolecular structures for the elaboration of a growing variety of materials.

### 2.1. Polysaccharides 

**Figure 2:**
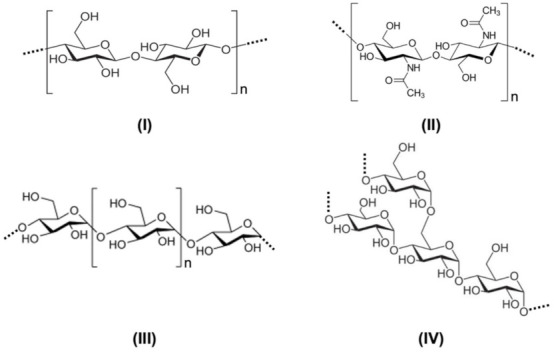
Molecular structures of cellulose (**I**), chitin (**II**), and of the two polysaccharides of starch, the linear amylose (**III**) and the branched amylopectin (**IV**).

Polysaccharides are natural polymeric carbohydrate structures, formed of repeating units of either monosaccharides (e.g., glucose, fructose, galactose) or disaccharides (e.g., sucrose, lactose) joined together by glycosidic bonds. Three of the most abundant natural polysaccharides are illustrated at the end of the topic in Figure 2, i.e., cellulose, chitin, and starch. Polysaccharides range in structure on the one hand, from linear to highly branched architectures and, on the other hand, from highly homogeneous in terms of monomer units (potentially crystallizable homopolymers such as cellulose, starch, and chitin) to macromolecules made up of different units, usually assembled in a random fashion (amorphous copolymers generically known as hemicelluloses) [6]. 

In general, the most common chemical modifications of polysaccharides are through their OH groups, with the primary moieties being more reactive than the secondary counterparts. Although there is an increasing interest in the exploitation of less conventional natural polysaccharides to the design of novel molecules with sophisticated architectures—which is especially the case of the microbial exopolysaccharides pullulan, alginates, curdlan, schizophyllan, among others [24], one can state that those that can be directly extracted from vegetable or animal biomass without the need of complex upstream and downstream processes are most likely to do the job in terms of large-scale production. 

In this regard, the focus is mainly directed to cellulose, the most abundant natural polymer on earth (~10^12^ t), made up of a regular linear semicrystalline structure of β-(1→4)-D-glucopyranose units in a ^4^C_1_ conformation (Figure 2I). Cellulose always appears in nature in the form of hollow fibers, and its supramolecular structure gives rise to the ongoing hot topic of nanocellulose, which integrates bacterial cellulose (BC), made of continuous nanofilaments of pure cellulose with diameters of 3 to 10 nm [25], nanofibrillated cellulose (NFC), with diameters and lengths of 5–50 nm and 0.2–2 μm, respectively, and cellulose nanocrystals (CNC), which are close to cellulose monocrystals, with diameters of 3–10 nm and lengths of 50–500 nm [25,26,27,28,29,30,31,32]. 

Cellulose nanofibers and cellulose nanocrystals are commercially available products obtained via chemical and mechanical treatments, including ball milling, high-pressure homogenization, 2,2,6,6-tetramethyl-piperidine-1-oxyl (TEMPO) oxidation, acid and enzymatic hydrolysis, and combined techniques [33]. Bacterial cellulose is produced from different genera, with fermentation conditions varying mostly with respect to the culture media [25]. Because of their very high water affinity coupled with large surface-to-volume ratios, nanocelluloses form self-sustained gels with as little as 2% solid and find applications in many fields [6]. Apart from the obvious possibility of remodeling the current food packaging scenario by taking advantage of the outstanding properties of nanocelluloses [34], recent contributions demonstrate that they appear as favorable candidates for the design of hydrogels and cryogels [35], including those that exhibit electric conductive properties [36], of gas barrier [37] and membrane filtration materials [38], of supercapacitors [33], of photoremediation agents for contaminated environments [39], of oil and gas production green additives, especially in enhanced oil recovery and hydraulic fracturing applications [40], and of novel biomedical systems, as for targeted chemo-protodynamic/photothermal cancer therapy [41]. Other common applications involve nanocelluloses as paper additives, implants, dentistry aids and cosmetics, and reinforcing elements in composite materials [42].

The surface modification of cellulose fibers is a common approach based on the abundance of accessible hydroxyl groups, which provide an excellent platform for covalent surface functionalization by conventional reactions that allow the insertion of ester, ether, carbonate, carbamate, and isocyanate groups, as well as polymer grafts, and the adjusting of hydrophilicity, hydrophobicity, dispersibility, and (bio)compatibility. Many possibilities for surface and bulk chemical modification of celluloses were reported in the literature over the last decades, mainly aiming at providing a compatible environment for fiber-matrix interaction in composites, and at preparing hydrophobic and oleophobic cellulose surfaces [6,43,44]. The surface chemical modification of cellulose has been widely considered, for instance, to improve the compatibility between hydrophilic cellulose and the bio-based, brittle, hydrophobic poly(lactic acid) [45]. A critical review published a couple of years ago by Meier and co-workers tackled an important feature of cellulose modification, i.e., the commonly exploited renewable origin of the raw materials, which is not always accompanied by chemical modification reactions that follow the 12 principles of green and sustainable chemistry [46]. The authors provide insights on sustainability assessments that might be taken into account for overall processes of reduced environmental impact. 

Some interesting new approaches to the modification of cellulose-based architectures involve, for instance, the combination of microcrystalline cellulose with maleic anhydride for an in situ fibrillation and surface modification through a solvent-free and low-energy pulse plasma polymerization [47], the use of biological catalysts for cellulose acylation/esterification mediated by hydrolases, for cellulose grafting mediated by laccases, and for cellulose phosphorylation mediated by hexokinases [48], and the chemical modification of cellulose nanocrystal reducing end-groups, which simply utilizes the reactive aldehyde to introduce functional moieties at the end of cellulose chains and enables particular assemblages relying on the asymmetric nature of the particles [49].

Starch deserves equal attention when considering the development of polysaccharide-based materials. It is made up of two macromolecular structures bearing the same glucose monomer units, viz., linear amylose and branched amylopectin (Figure 2, III and IV, respectively), that allow, on the one hand, a wide array of possible chemical modifications, and on the other, the production of thermoplastic starch (TPS), a cheap biodegradable material with numerous potential applications [6]. Starch plasticization is a process based on the transformation of the semi-crystalline starch granules into a homogeneous continuous material in the presence of an appropriate plasticizer and water while applying sufficient thermomechanical energy [50,51]. It is noteworthy that the use of plasticizers with varying functional groups, molecular weights, thermal stability, and compatibility, interferes on the starch–plasticizer molecular interactions and, therefore, on the properties, retrogradation, and processing of TPS obtained by extrusion, which was the subject of a comprehensive review recently published [52].

The research on starch-based materials is mostly based on the design of novel systems—mainly blends and composites—aiming at overcoming some practical drawbacks of TPS, whose implementation has been limited by its tendency to absorb moisture and thus a loss of mechanical properties. The efforts devoted to the association of TPS with natural montmorillonite, organically modified montmorillonite, cellulose nanocrystals, and cellulose nanofibers were discussed [53], with particular attention directed to the ensuing properties of composites, such as biodegradability, mechanical, barrier, and optical properties. In fact, the association between starch and (nano)celluloses has been vastly exploited in recent literature, and some approaches include the incorporation of starch into non-renewable polymers aiming at reducing the dependence on petroleum-based macromolecular materials. In this context, cellulose nanocrystals were added to TPS and the mixture was blended in an extruder with LDPE at various loading levels, and the materials bearing 1% of cellulose nanocrystals with respect to LDPE/TPS blends exhibited improved strength and barrier performances [54]. All-renewable TPS-based blends also deserve attention and may be obtained via different processing techniques. The in situ chemical grafting of poly(lactic acid) and TPS, using glycidyl methacrylate in the presence of benzoyl peroxide as free radical initiator, was described in a two-step procedure of twin-screw extrusion followed by an extrusion blown film process, which led to final blends with improved elongation (up to 144%) and oxygen permeability (up to 41%) with respect to virgin PLA films [55]. The association of natural rubber and TPS is of interest, but most publications available so far describe poor mechanical properties of the final materials essentially due to the inefficient compatibilization between both phases. A recent contribution of Cai and coworkers tackled this issue by inserting epoxidized natural rubber as a compatibilizing agent, which led to materials of good mechanical properties [56]. The influence of plasticizer type and content on the properties of TPS is also an object of investigation, which was the case for TPS blown films plasticized with bio-based xylitol and sorbitol, whose tensile strength, stiffness, and water vapor and oxygen barrier properties were superior when compared with those of the corresponding TPS prepared solely with glycerol [51].

Finally, chitin is widely exploited as an abundant animal polysaccharide, made up of N-acetyl-D-glucosamine units linked by β-(1-4) glycosidic bonds (Figure 2, II). With respect to the chemical structure of cellulose, in chitin one secondary –OH group is replaced by a methylamide function, which gives a very high cohesive energy that limits its processing. However, the straightforward conversion of chitin to chitosan (poly(β-(1–4)-2-amino-2-deoxy-β-D-glycopyranose) through the base-catalyzed hydrolysis of its amide functions is, up to the present, the most important utilization of the former in terms of industrial possibilities. Chitosan finds applications in pharmaceutics, biomedicine, packaging, wastewater treatment, cosmetics, food science, among others [6,57,58]. A comprehensive review of Acosta and coworkers was recently issued [59], and detailed information on the technological properties (such as solubility and viscosity), chemistry, and biological activities (such as antimicrobial, antioxidant, and anti-inflammatory activities) of chitosans are available. The authors additionally reviewed the use of chitosans in the green synthesis of metallic nanoparticles, as support for biocatalysts, and as versatile materials that can be exploited for the design of novel drug delivery systems. The state-of-art is complete when one mentions thiolated-chitosans as tunable materials that possess outstanding properties such as improved adhesion to biological surfaces, adjustable crosslinking and swelling, controllable drug release, enhanced permeation, and cellular uptake, efflux pumps and enzymes inhibition, metal ions complexation, as well as antioxidative properties, and radical scavenging activity [60].

### 2.2. Lignin

**Figure 3:**
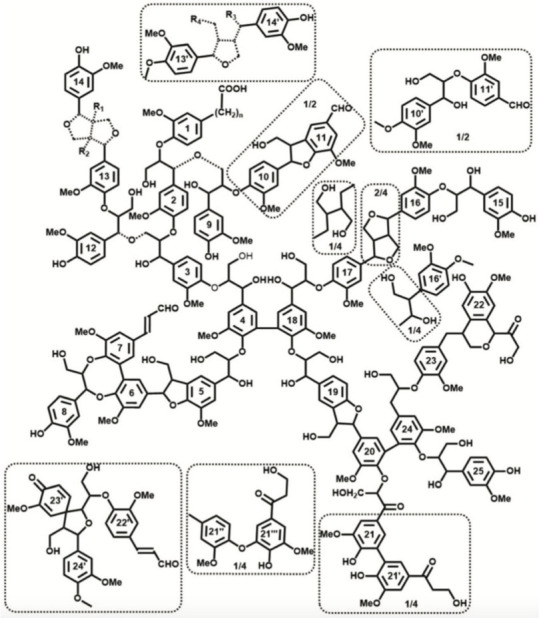
Tentative structural model of spruce milled wood lignin (MWL). Reproduced with permission from [63].

Lignin occurs throughout the vegetable realm and dominates woody morphologies together with cellulose and hemicellulose, where it plays the role of a hard matrix, moisture barrier, and resistance to microbial attack, contributing as well to biomass recalcitrance [6,61,62]. It may be defined as an irregular, oxygenated p-propylphenol supramolecular structure, formed by free radical polymerization of monolignols [63]. Understanding the precise molecular structure of lignins is still a work in progress, which is of primary importance not only for fundamental reasons but also to access lignin reactivity and valorization [63]. Recent studies enabled a deeper understanding of the molecular structure of spruce milled wood lignin (MWL) [63,64]. The authors proposed a very convincing rather branched and crosslinked structure (Figure 3) and mentioned that lignin in situ is much more difficult to analyze, but such analysis of model MWL may provide useful structural information that is inaccessible for the former. It is important to emphasize that the detailed structure of lignin varies from species to species and as a function of the age of a tree, although the presence of both phenolic and aliphatic hydroxyl groups, as well as of the typical “C9 motifs,” consisting of an aromatic ring joined by three aliphatic carbon atoms, are common to all lignins [6].

Although the amount of lignin extracted in pulping operations around the world is estimated at over 70 Mt per year, only about 2% is exploited as a source of chemicals or materials. The rest is burned as a source of energy for the process and for the regeneration of the pulping catalysts [6]. The consolidation of more sustainable and competitive biorefineries depends largely on the effective valorization of lignin, which can be explored as a source of chemicals and materials [38]. In fact, increasingly sophisticated processes are based on routes that enable maximum use of the individual components of plant biomass, which can be exemplified by the recently published work on the oxidative fractionation of lignocellulosic biomass, conducted in the presence of Co-N-C catalysts and O_2_ in acetone as a solvent, which allowed high recovery efficiency of phenolic aldehydes and carboxylic acids (vanillin, syringaldehyde, p-hydroxybenzoic acid, vanillic acid, and syringic acid), with the simultaneous preservation of the cellulosic fraction [64].

So far, the methods commonly used to convert lignin into compounds of interest generally follow the sequence (i) fractionation of lignocellulosic biomass (pulping), (ii) lignin depolymerization, and (iii) synthesis of specific chemicals. Different fractionation processes are known, carried out from different reaction mechanisms. However, in general, delignification occurs mainly through the cleavage of ether bonds present in native lignin, which releases lignin fragments that remain in solution (black liquor). These fragments react with each other by condensation reactions, forming carbon–carbon bonds that are hardly cleaved [65,66]. Thus, after the fractionation process, the lignin extracted from the biomass displays a different chemical structure from the natural lignin, being more complex, more recalcitrant, and composed of several lignin derivatives with individual concentrations not exceeding 1% [67].

Studies that take into account the development of precise methods to expand the application possibilities and the production of aromatic compounds directly derived from renewable sources are of great interest. In this sense, pulping methods were proposed in which lignin fragments are catalytically converted soon after their release from the lignocellulosic matrix. These processes are known as pulping with a “lignin-first” approach, a term that indicates that the valorization of lignin is prioritized over the valorization of carbohydrates [68,69]. An alkaline aerobic oxidation method that converts lignin into a collection of oxygenated aromatics, including vanillin and p-hydroxybenzoic acid, was recently published, and centrifugal partition chromatography was proven effective to isolate the individual compounds from the complex product mixture [70]. In all cases, a careful choice of the biomass fractionation process, ideally avoiding extensive side reactions of lignin, allows the production of promising aromatic building blocks for polymer synthesis.

In general, the exploitation of lignin fragments to prepare macromolecular materials consists of three main approaches, namely (i) their use as blend components, (ii) their direct use as macromonomers in polycondensation reactions or to prepare carbon fibers, and (iii) their chemical modification prior to their intervention in the synthesis of a polymer [6]. The preparation of renewable aromatic polymers represents a challenge in polymer science due to the remaining obstacles related to the abovementioned isolation and purification of aromatic compounds from lignin [71,72]. Parit and Jiang [73] recently published a review paper contemplating multiple strategies for the production of thermoplastic polymers from lignin. The authors described several possibilities for chemical modification of lignin, which are often conducted with the aim of controlling its homogeneity, reactivity, processability, and compatibility for the synthesis of thermoplastic copolymers and for the processing of polymer blends, in addition to lignin copolymerization methodologies such as “grafting from” and “grafting onto”. The contribution of Grossman and Vermerris [74] also included the use of lignin as a basis for the production of nanomaterials, with potential application in electronic and biomedical devices. In these and other similar works, the conversion of lignin to polymers does not involve the isolation of aromatic monomers, given the complexity of the starting material. In fact, economically viable exploitation of lignin in macromolecular materials still implies inevitably the use of fragments of its natural macromolecular architecture, that can vary considerably both in molecular weight (from ~1 to ~100 kDa) and specific structure, with glass transition temperatures (Tg) ranging broadly from 70 to 180 °C [6]. 

### 2.3. Natural Rubber

Natural rubber (NR) is a hydrocarbon polymer of high molecular weights (10^4^ Da < MW < 10^6^ Da), naturally available as a colloidal dispersion (latex) that may be extracted from more than 2500 plant species, although the *Hevea brasiliensis* tree deserves greater attention. The “bled” latex mainly consists of *cis*-1,4-poly(isoprene) particles surrounded by a thin layer of proteins, lipids, and long chains of fatty acids, which gives the particle a negative charge and ensures the colloidal stability of the medium. Due to the unique physical and chemical properties of NR, including resilience, elasticity, resistance to abrasion and impact, efficient heat dispersion, and malleability at cold temperatures, it is an industrially important polymeric material used to manufacture adhesives, tires, surgical gloves, health equipment and accessories, balloons, tubes, amongst others. The global consumption and production of NR in 2017 exceeded 1.3 million tons, mainly because, even though a large number of synthetic polymer counterparts are available now, only a few possess physical properties that are comparable to those of NR [75,76]. Nevertheless, the related production process is still manpower-intensive, decreasing its attractiveness whenever economic growth leads to demand for higher wages [77].

Apart from the technological advances related to the improved performances of the numerous NR artifacts, the scientific interest has been mainly devoted to related composite with nanofibers, blends, and reversible crosslinking [6]. In general, the incorporation of unmodified natural fibers as fillers for the NR matrix does not provide the desired reinforcement of the ensuing composites, and several surface modification methods can be introduced to improve the overall performances of the final materials. Roy and co-workers recently reviewed the present status and prospects of such materials, including a section devoted to the most promising applications in the building, construction, packaging, and automotive industries [78]. Another equally important approach is the modification of natural rubber, which can be carried out by tuning nano- and microstructure and/or composition of latex particles by surface chemical functionalization, structural modification, and incorporation of inorganic nanoparticles [79].

Recent investigations have considered the utilization of NR in progressively more sophisticated materials. Thermal conductive and low dielectric constant composites were designed via functionalization of boron nitride platelets with poly(dopamine) and γ-methacryloxypropyl trimethoxy silane, their incorporation into NR, and vulcanization at 150 °C, leading to materials with good potential to be applied in electronic devices and long-term operation capacitors [80]. As natural rubber-based products are often exposed to harsh environments, much has been done to develop flame-retardant materials by incorporating flame-retarding units into the polymer chains (in the polymer backbone or as side chains) or by adding flame retardants into the NR matrix by physical blending [81]. The pavement industry can be largely beneficiated from natural rubber, as it can be used as a bitumen modifier to prolong the lifespan of flexible pavements while aggregating environmental sustainability [82]. Finally, NR displays many properties that are compatible with the biomedical field, as in vitro biocompatibility, peptide- and angiogenesis-inducing activity for tissue repair, suitability for guided bone repair (useful for the regeneration of bones and teeth), as well as the ability to serve as a platform for the controlled delivery of active ingredients [75]. Other investigations related to the chemical modification of NR will be addressed again in the section dealing with furans and their aptitude to go through the thermo-reversible Diels–Alder reactions. 

## 3. Bio-Based Routes for Commodity Polymers 

The possibility of producing *the exact same monomers* that are classically obtained from petroleum using, instead, a fully renewable platform, has recently received much attention, since the well-established production—processing—demand chain of the polymeric materials is allied with the current need for the overall reduced carbon footprint. Recently, Siracusa and Blanco [83] published a broad review article on the advances in the research and development of bio-based polyethylene (PE), polypropylene (PP), and polyethylene terephthalate (PET), that are amongst the most important polymeric materials for packaging and engineering applications in terms of annual production volume. 

Up to the present, the main case of success inserted in this context was achieved by the Brazilian Braskem Company that, since 2012, produces several hundred thousand tons of PE of different grades, including copolymers, from sugarcane juice. The process is based on the catalytic dehydration of ethanol to ethylene, using solid metal-oxide catalysts. The polymerization steps are identical to those employed for the fossil-based ethylene, and the final products are therefore also identical. Terms such as “green-PE” or “bio-PE” must be used carefully and responsibly, as it may give consumers the wrong idea of an environmentally innocuous product, when it is definitively not. However, the case is a very strong proof of concept that bio-based polymers are profitable and have commercial demand. The production of poly(propylene) (PP), in which ethanol is converted to propylene through metathesis of ethylene with 2-butene, is also being investigated, but the process still requires further optimization in order to improve its economic competitiveness compared with the very efficient petrochemical alternative [6]. In fact, a techno-economic evaluation of bio-based propylene production in a Brazilian sugarcane biorefinery was conducted a few years ago [84], and, even considering a minimum bio-propylene selling price, the results indicated final prices up to 90% higher than those of the conventional fossil-based product. However, when considering additional aspects as, for instance, policies that would result in lower capital costs, a competitive price could be achieved. The production of a truly bio-based PET must consider the origin of both starting chemicals, i.e., terephthalic acid and ethylene glycol. In both cases, different options of synthesis are already available, mainly based on chemical routes in the case of the former, and on fermentative processes in the case of the latter. A thorough monograph dealing precisely with the many possibilities of accessing bio-based PET is available [85].

It is also worth mentioning that the possibility of preparing bio-based polyamides of commercial interest. The production of succinic acid, 1,4-diaminobutane, and 1,5-diaminopentane by fermentation of metabolically engineered bacteria was reported, which were used to synthesize fully renewable nylon-4,4 and nylon-5,4 that carry the additional advantage of potential biodegradability [86]. Recently, biobased aromatic building blocks were synthesized from methyl coumalate, which is obtained from the dehydrative, decarbonylative dimerization of the glucose fermentation product malic acid, and used to prepare (semi-)aromatic polyamides [87]. 

Other important bio-based monomers may be synthesized to mimic those traditionally coming from fossil feedstocks. Acrylates and methacrylates are increasingly gaining attention in this context, as they can be prepared from lignocellulosic-derived phenolic compounds, terpenes, lactic acid, isosorbide, lactone-based acrylates, glycerol, levoglucosenone, vegetable oils, and fatty acids, among other renewables [88].

## 4. Broadening the Horizon: Bio-Based Routes for Original Polymers

The polymerization of (macro)monomers that are exclusively obtained from renewable resources offers the great advantage of exploiting a broad class of raw materials to produce novel structures and architectures that may not be straightforwardly prepared from the fossil platform. This approach provides access to a new generation of macromolecular materials that may greatly contribute to the establishment of viable biorefineries in a near future. The subject is evolving quickly in many contexts and, since numerous textbooks and monographs are available on this wide topic, only the most recent and promising contributions will be tackled here.

### 4.1. Vegetable Oils and Glycerol

**Figure 4:**
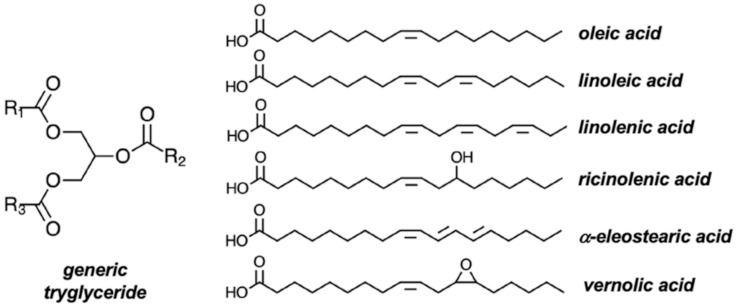
Molecular representation of a generic structure of natural triglycerides, with R_1_, R_2_, and R_3_ representing fatty acid chains; the most common naturally available fatty acids are depicted on the right.

Vegetable (or plant) oils are a huge family of natural viscous liquids sharing a common triglyceride molecular structure (Figure 4), in which R_1_, R_2_, and R_3_ correspond to fatty acid chains whose precise chemical nature vary widely depending on the source from which they were extracted. According to the United States Department of Agriculture [89], almost 90% of all vegetable oil that is produced and distributed around the world come from soybean (*Glycine max*), palm (*Elaeis guineensis*), rapeseed (*Brassica napus*), and sunflower (*Helianthus annuus*) seeds, with oleic and linoleic acids as their major fatty acids, followed by palmitic, stearic, and linolenic acids. Up to 20 different fatty acids, with 14–22 carbon atoms, have already been identified in appreciable quantities in vegetable oils [6]. As vegetable oils are vastly used for food and feed, as well as for energy generation and for the production of chemicals and materials, they are often classified as the most important renewable feedstocks of the chemical industry.

The main characteristics that determine the physicochemical properties of vegetable oils are the length of the fatty acid chains, the stereochemistry of the double bonds, and the degree of unsaturation, with particular emphasis on the latter. The so-called drying oils are those that are unsaturated enough to form films when exposed to atmospheric oxygen and sunlight, being therefore mainly used in paints and coatings. This free radical crosslinking oxypolymerization involves the initial abstraction of a hydrogen atom from the methylene group positioned next to a double bond, which leads to the formation of peroxy radicals. In a later step, the recombination of radicals produces crosslinks (alkyl, ether, or peroxide). The degree of unsaturation is, therefore, a factor of considerable importance, since the double bonds present along the chains are the promoters of many possible functionalization reactions for the chemical industry.

For decades, vegetable oils were preferred for polymer synthesis among other bioresources due to their abundance and democratic geographical distribution, low price, easiness of chemical modification of the triglyceride structures, and potential biodegradability of the final materials. A book fully devoted to the production of polymers from vegetable oils was published a few of years ago [90], however, as it is a hot topic in the field of polymers from renewable resources, an update seems convenient and is available for the interested reader [91]. In fact, mostly in the past two decades, the development of novel polyesters, polyurethanes, polyamides, polycarbonates, UV-curable coatings, among many other materials based on vegetable oils, has been described [90,91,92]. The strategy may involve, on the one hand, the preparation of highly branched and crosslinked materials, as a result of exploiting the polyfunctional nature of crude triglycerides, and on the other, the breakdown of triglycerides into the corresponding fatty acids prior to the synthesis of linear materials. 

The direct polymerization of polyfunctional vegetable oils into thermosets is a possibility that avoids preliminary synthetic steps and therefore represents a more sustainable approach towards polymeric materials [93]. The 1,2-disubstituted C=C unsaturation of common fatty acids often react slowly in the presence of free radical and cationic initiators, and this strategy demands an association with a more reactive comonomer such as styrene and divinylbenzene, or a preliminary step for converting triglycerides into more reactive monomers, such as epoxidation or thiol-ene reactions [90,91]. The epoxidation of vegetable oils corresponds to a routine industrial process employed, for example, for the synthesis of plasticizers for several commodity polymers, but it is also a useful platform for the design of novel macromolecular materials. This is a versatile approach, as epoxy thermosets based on vegetable oils may be produced with a very broad range of properties depending on the specific raw material, reaction characteristics, and comonomers employed. Allasia and coworkers described the synthesis of thermosets based on epoxidized soybean oil, succinic, adipic, and sebacic acid, and a fatty acid-based dicarboxylic acid [94]. The fully renewable thermosets were characterized, and their potential biodegradability was assessed in alkaline and compost conditions. Considering the most recent contributions available in the literature, one can observe that there is an increasing tendency to go beyond the synthesis of plant oil-based thermosets, and to also evaluate the post-use properties of the final materials. Thermally and chemically reversible dynamic covalent networks were obtained by crosslinking epoxidized soybean and linseed oils with 2,2-dithiodibenzoic acid, a disulfide-containing carboxylic diacid hardener that allows chemical recyclability and thermal reprocessing [95], and the work was complemented with a further investigation of many other vegetable sources as raw materials for the same purpose [96]. Vitrimers are a class of resins that can change their topology via a dynamic bond exchange, resulting in their self-healing and reprocessable molding [97]. Vegetable oil-based epoxy vitrimers with triple exchange crosslinkers of carboxylate ester bonds, disulfide bonds, and dioxaborolane were prepared and exhibited self-healing and recyclability properties, enabled by the dioxaborolane metathesis reaction of boronic ester, which is activated at room temperature with moderate humidity [98]. 

The use of pristine and chemically modified triglycerides as monomers that possess their multifunctional nature preserved is an important strategy for the preparation of original cross-linked materials with lower Tg, with respect to other commercial resins based on the fossil platform. However, hydrolysis and transesterification reactions are more frequently conducted for the isolation of fatty acids (or of their corresponding fatty esters) and further preparation of linear, hyperbranched, and cross-linked materials [91]. In fact, the concept of producing linear polymers from plant oils started in the 1950s and has witnessed an impressive evolution in the past few years. One can mention, for instance, many possible routes to convert fatty-acid-based monomers into polyolefins with the most diverse pendant groups along the polymer chain via radical polymerization [99]. Nomura and Awang reviewed the many contributions available based on the synthesis of 1,ω-fatty acid diesters and diols, which can be used as monomers for the synthesis of long-chain aliphatic polyesters by numerous routes [100]. A facile route for semi-aromatic polyamides from dimethyl 9-octadecenedioate and *p*-xylylenediamine or diethylenetriamine was reported, leading to materials with good thermal and mechanical properties [101]. 

At this point, it is worth mentioning the increased attention that has been directed to tung oil, a non-edible, high-iodine value oil, perfectly suitable for polymer synthesis. Despite the readiness of being converted to surface-dry films given by its prompt susceptibility to radical oxydopolymerization, tung oil can be homo- and copolymerized, in a strategy that was first described in the late 1960s and again in the early 1980s [102]. In the last years, however, the number of related publications increased considerably, and some relevant examples involve the preparation of vitrimers [99], UV-led curable [103] and antifouling [104], self-healing smart coatings [92,105], and tung oil/furfuryl alcohol networks [106], designed following a previous investigation on the cationic polymerization of tung oil and methyl α-eleoestearate [107]. 

Many other equally important investigations are continuously being published, and it is expected that thermoplastic and thermoset materials based on plant oils may reach the market progressively.

**Figure 5:**
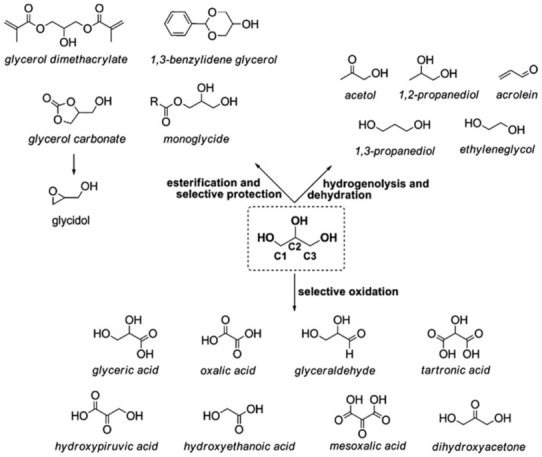
Conversion of glycerol into value-added compounds via different catalytic pathways [6].

In industrial processing, vegetable oils are converted to glycerol plus fatty acids, methyl esters, and fatty alcohols, and subsequent chemical modification steps are often conducted to obtain the specific chemicals of interest [91]. Taking the biodiesel industry into account, the search for novel high value-added products based on glycerol is a topic that deserves attention, mostly because its industrial surplus has induced an unneglectable market saturation. Glycerol ethers, for instance, meet the renewable green solvent requirements and have attracted a great deal of attention in various applications [108]. Glycerol microbial biotransformations, mainly into dihydroxyacetone, are also in the spotlight [109]. In fact, glycerol is a versatile compound susceptible to a large number of (bio)chemical conversions, leading to very interesting building blocks for the preparation of a large family of chemicals (Figure 5). Besides glycerol itself, many of its derivatives are also suitable for polymer synthesis, and this possibility has been stimulating academic and industrial investigations [90,110,111]. Dihydroxyacetone, glyceric acid, hydroxypyruvic acid, mesooxalic acid, and tartronic acid, for instance, have great potential as intermediates for polymer synthesis [6].

With respect to glycerol-based polymeric materials, apart from the investigations related to the production of oligomers that find applications in the food, pharmaceutical, cosmetic, and polymer industries [6], other interesting approaches have recently indicated that the prospects are good, and the field might broaden in a near future. In general, polyglycerols are produced either from epichlorohydrin, classified as a probable carcinogen in humans, or directly, in the presence of a strong base. The search for non-toxic counterparts is of particular interest for the cosmetic and food sectors. A review article published by Ebadipour and coworkers indicates that alkaline homogeneous and heterogeneous catalysis might be strategic routes to yield glycerol-based polymers, as it offers higher glycerol conversions, but some practical issues remain to be solved [112]. Another possible approach is to synthesize glycerol carbonate from crude glycerol and urea, which can be then converted to polycarbonates, polyglycerol esters, hyperbranched polyols, and non-isocyanate polyurethanes using zinc, magnesium, tungsten, and ionic liquid-based catalysts [113]. It is also important to mention the initiatives that aim to design overall sustainable processes, which are normally achieved by employing mild reaction conditions and/or biological catalysts. Poly(glycerol sebacate) with varying molar ratios of glycerol, sorbitol, sebacic acid, 1,6-hexanediol or 1,8-octanediol, were synthesized using *Candida antarctica* lipase B (CALB) [114], yielding semi-crystalline materials that could be processed by electrospinning and exhibited potential to be applied in the biomedical field. Smart polymeric materials can be also produced from glycerol, which was the case of a pH-responsive dendrimer, prone to encapsulate or to release dyes depending on the pH. The synthesis consisted of the reaction of glycerol with acryloyl chloride, followed by the coupling of the ensuing glycerol triacrylate with ethylenediamine, the production of a glycerol-based dendrimer via free radical mini-emulsion polymerization, and film casting [115]. A new class of (meth)acrylate polymers was designed from glycerol ketals, whose syntheses were carried out with glycerol and ketones of varying specific molecular structures [116]. A broad study evaluated the cradle-to-gate life cycle assessment of glycerol-based adhesives produced through a reversible addition-fragmentation chain transfer polymerization process, and the results indicated lower environmental impacts with respect to the fossil analogs [117]. Such initiatives are important to understand the overall impacts of bio-based materials.

### 4.2. Furans

**Figure 6:**
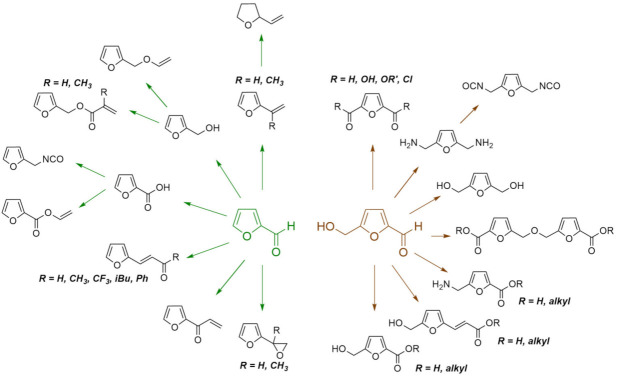
F (in green) and HMF (in brown) as typical precursors to some furan monomers that can be polymerized by chain-growth and step-growth reaction mechanisms, respectively.

The exploitation of the furan platform has become one of the most relevant aspects of the renewable resources paradigm within the context of polymer chemistry. A recent search on Web of Science, using the keywords “furan” and “polymers”, and further analysis of the results showed an impressive increase of more than 350% in the number of yearly publications during 2011–2020 with respect to the previous decade (2001–2010). The strategy relies on the exploitation of two furan derivatives readily available from simple chemical transformations of natural polysaccharides: pentose- and hexose-containing polysaccharides constitute, respectively, the main source of furfural (F) and 5-hydroxymethylfurfural (HMF) [118], which are the precursors of a multitude of monomers that have been successfully synthesized, characterized, and polymerized [6,16,119,120,121,122,123] (Figure 6). It is important to mention that, although the industrial production of furfural is fully consolidated (estimated at 300 kt/year, costing approximately USD 1/kg), the search for improved industrial processes to produce 5-hydroxymethylfurfural is of great interest. Moreover, environmentally friendly and energy-saving methods for the conversion of furanic compounds, e.g., via photocatalysis [124] and fermentative processes [125] are in full development and may offer new paths for the production of the corresponding value-added products. 

Two main strategies are possible to produce polymers from furan-based building blocks, viz., the synthesis of monomers that are suitable for chain (co)polymerizations, forming macromolecules with pendant furan moieties, and of those that are suitable for step-growth polymerizations, which produce macromolecules with the furan heterocycle within their backbone [6]. The former strategy is mostly based on F, which is an important platform for the synthesis of unsaturated furans (Figure 6, left part), whose susceptibility to polymerize via chain reactions depends on their specific structure, and on their actual response to different types of initiation. The latter strategy, on the other hand, relies on the possibility of converting 5-hydroxymethylfurfural into bifunctional building blocks (Figure 6, right part), which opens the way for the preparation of polyesters, polyamides, polyurethanes, and other polycondensates that have been synthesized from petrochemical precursors since the beginning of last century but derived instead from precursors which are based on vegetable renewable resources. It is important to emphasize that the range of possibilities is not limited to the structures presented in Figure 6, and efficient routes for novel derivatives are constantly being reported. This is the case of furandiacylazide, from which the corresponding furanic diisocyanate can be obtained with yields up to 99% [126], and bisfurfural, that may serve as a platform for original monomers and polymers [127]. 

The three main routes to furan-based chain polymers (i.e., via free radical, cationic, and anionic polymerization) exhibit specific behaviors due to the dienic character of the furan ring, that influences the stability of intermediates formed after a homo- or heterolytic cleavage of conjugated double bonds. Although only a modest number of furan monomers respond adequately to free radical initiation (as in the case of furfuryl acrylate and methacrylate), ionic polymerization may be considered and sometimes yields interesting macromolecular materials. Moreover, 2-Alkenylfurans (2-vinylfuran, 2-vinyl-5-methylfuran, 2-isopropenylfuran, and 2-isopropenyl-5-methylfuran) are readily activated by even the mildest cationic initiators because of their pronounced nucleophilic character; the anionic polymerization of 2-furyloxirane is possible due to the high reactivity of the oxirane ring towards strong nucleophiles [119].

With respect to polycondensation mechanisms, attention must be given to the conversion of furfuryl alcohol into thermosets, since they correspond to widely exploited materials in a metal foundry, refractory bricks, polymer concretes, porous carbonaceous materials, industrial electrodes, coatings for aggressive environments, and underwater wood protection [6]. More recent studies employ them as matrixes in composites reinforced with various fillers and nanoparticles, sol-gel-based nanocomposites, as well as a comonomer for the preparation of interpenetrating networks [106].

The investigations have been more active, however, in the exploitation of the bifunctional furan-based monomers that can be prepared from 5-hydroxymethylfurfural, with a focus on 2,5-furan dicarboxylic acid, the sustainable heterocycle counterpart of terephthalic acid that may serve as platform for the preparation, for instance, of polyesters [128] and polyimides [129] with considerably reduced environmental impact. This is where poly(ethylene 2,5-furandicarboxylate) (PEF) stands, which is now in process of being scaled up to industrial levels as a (often better) replacement of PET. The synthesis of poly(butylene 2,4-furandicarboxylate) was also demonstrated, and the material exhibited excellent gas barrier properties, thermal stability, flexibility, and toughness [130]. With the aim of describing a fully renewable polymeric material, cyclic oligomers based on 2,5-furan dicarboxylic acid and isomannide, a sugar-based structure to be tackled in the next section, were considered a few years ago [131]. Specifically, isomannide 2,5-furandicarboxylate was polymerized with oligo(butylene 2,5-furandicarboxylate) at 220 °C with Sn(Oct)_2_ as a catalyst, yielding random copolyesters of average molecular weights up to 50 kDa.

**Figure 7:**
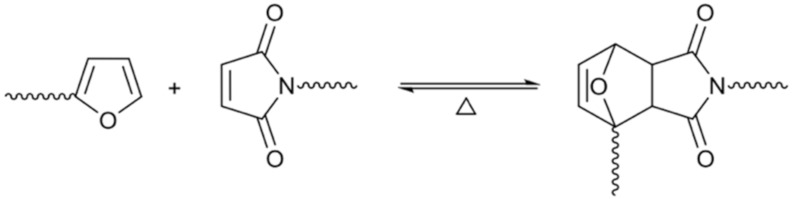
The DA equilibrium governing the polycondensations of furan and maleimide end-groups.

Undoubtedly, the most significant contribution to furan polymers in recent studies is related to the application of the *click* Diels–Alder (DA) reaction to step-growth polymerizations involving monomers or polymers incorporating both furan (F, diene) and maleimide (M, dienophile) functions [6,132]. This combination is particularly suited to the synthesis of macromolecular materials that can be readily recycled, possess self-mending properties, and can alternatively be converted into heat-resistant macromolecules by turning the thermally sensitive DA adduct into an aromatic structure [133]. The DA reaction responsible for this reversible growth is shown in Figure 7, where the forward reaction giving the DA adduct predominates up to about 60 °C, whereas the equilibrium is heavily shifted in favor of the retro-DA (rDA) reaction that deconstructs the couplings above about 110 °C. An important restriction of the reaction was known to be related to the use of electron-rich furans as dienes, such as furfurals and their oxidized variants, furoic acids. However, this was recently overcome by demonstration of the susceptibility of 2-furoic acids, as well as their esters and amides counterparts, towards Diels–Alder reactions with maleimides [134]. The strategy goes beyond, as the DA reaction between furan and maleic anhydride allows the production of low molecular weight chemicals, such as phthalic anhydride [135].

In general, the three main routes to DA-based furan polymers are (i) linear polymerizations involving either FF and MM, or MF monomers, (ii) hyperbranching or crosslinking reactions with monomers with functionality higher than two, and (iii) crosslinking reactions of a polymer bearing pendant F (or M) moieties with a bismaleimide (or difuran) coupler [6,132]. The many investigations devoted to this specific reaction published so far provided enough understanding of the systems, allowing the progressive incorporation of the designed materials in fine applications, such as in drug delivery, bio-scaffolds, diagnostics, and self-healing biomaterials [136], in the production of thermo-reversible networks based on plant oils, natural rubber, starch, chitosan and cellulose [137,138,139,140,141], and for the synthesis of copolymers that cannot be accessed by conventional processes [142]. After more than 50 years of intensive and systematic studies on furan-based polymers, opportunities are still wide open to gather more precise mechanistic information and to produce original macromolecules of broad interest.

### 4.3. Sugars and Terpenes

The possibility of making polymers using carbohydrate-based and terpene-based monomers has become a relevant research and development activity within the field of polymers from renewable resources. The former strategy is based on the large variety of sugars that can be readily obtained from edible and non-edible crops, which opens the way for the preparation of a very broad family of low-cost building blocks.

**Figure 8:**
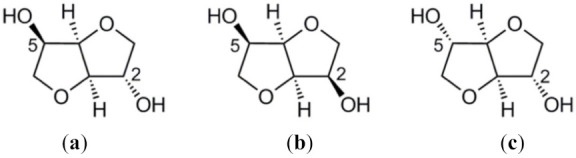
Stereo-isomeric forms of isohexides: isosorbide (**a**), isomannide (**b**), and isoidide (**c**).

A monomeric unit of carbohydrate (glucose or mannose) can be hydrogenated to produce sorbitol and mannitol. Subsequent double dehydration of sorbitol and mannitol leads to three main 1,4:3,6-dianhydrohexitol isomers (isohexides), namely isosorbide, isoidide, and isomannide, differing on the relative position of the two hydroxyl groups with respect to the “V-shaped” tetrahydrofuran dimer (Figure 8) [143]. The application of these and other sugars monomers in polymer synthesis was thoroughly discussed in a comprehensive review published by Galbis and coworkers a few years ago [144] and recently updated with a particular focus on pharmaceutical applications [145].

Although the three diols depicted in Figure 8 may be exploited as a source of monomers and polymers, isosorbide is the most relevant, participating as such or after appropriate chemical modifications, in the synthesis of macromolecular materials with very diverse properties [146]. A very complete monograph on the topic discusses some important features of this approach, mainly dealing with different polymerization routes that have been developed in the last years to overcome the challenges associated with the direct use of isosorbide in step-growth polymerizations [147]. In general, those challenges are the result of the rather low reactivity of the -OH groups, leading to low molecular weight materials (Mn ≤ 10 kg mol^–1^), with isosorbide contents limited to 50 mol% to avoid the need for extreme reaction conditions. Among the new synthetic strategies, one can mention a very smart one based on the synthesis of a tricyclic ether from isosorbide, further submitted to cationic ring-opening polymerization [148]. The approach allowed to control the polymer architecture, which cannot be done via polycondensation. Isosorbide monoepoxides were polymerized via anionic ring opening [149]. Isosorbide dimethacrylates were synthesized with *p*-, *m*-, and *o*-substituted aromatic spacers, specifically with the purpose of replacing bisphenol A in dental applications [150].

The greater deal of attention that has been directed to isosorbide is mostly because, among the three isomers, it is the only one that is an industrial commodity, available at reasonable prices from starch. Some progress is being reported to establish economically viable routes for the other analogues, considering the remarkable potential they offer to the design of macromolecular materials. The efficient and scalable production of isoidide from isosorbide was reported [151], which is a significant step toward the commercial production of this building block. The approach was based on a selective lead(II)-catalyzed acylation of the endo hydroxyl groups present in isosorbide and isomannide, followed by separation of the acylated material from unreacted isoidide by continuous extraction.

Other synthetic sugar-based monomers (such as xylitol, erythritol, mannitol, galactitol, among others) are also exploited through their various -OH functionalities, and chemical modification routes are considered for the preparation of diols, dicarboxylic acids, diester, diamines, diisocyanates, hydroxyacids, lactones, aminoacids, lactams, and the ensuing polyesters, polyethers, polycarbonates, polyamides, and polyurethanes, in the form of both homo- and co-polymers. As for their good biocompatibility potential, isohexide-based polymers are often considered to be applied in biomedical systems [144]. However, other applications may be possible, mainly due to the stiff character of such structures, which allows tuning the properties of the final materials as a function of the comonomer used in their polycondensations [6,143,144,145,146].

The exploitation of the volatile portion of the distillation of the resin exudate of conifers is particularly interesting, as it offers a rich source of renewable unsaturated hydrocarbons. Commonly known as turpentine, it corresponds to a complex mixture, mainly composed of monoterpenes, which are extraordinarily diverse, not only because of the variety of their basic skeletons, but also because many stereoisomers are possible given the presence of stereogenic centers in every skeleton, and because of the wide variety of oxygenated derivatives (alcohols, aldehydes, ketones, and carboxylic acids) that can be derived from those basic skeletons [152]. In general, however, the major components of turpentine are α-pinene (45–97%), β-pinene (0.5–28%), and smaller amounts of other monoterpenes. Limonene, a commercial monoterpene that can be isolated from citrus fruits, also deserves attention as it finds applications in food, pharmaceutical, and cosmetic products. 

(Co)polymerization reactions involving natural terpenes have been essentially limited to β-pinene via cationic and free radical mechanisms, leading, however, to low-molecular-weight products due to the occurrence of transfer reactions that can only be minimized under specific conditions [6]. The more abundant α-pinene, on the other hand, is rather less reactive for its endo-cyclic double bond [152]. The functionalization of monoterpenes is very promising to increase their potential as monomers. A metal-free conversion of α-pinene into δ-pinene was found to be useful to synthesize polyolefins via ring-opening metathesis polymerization using Grubbs 3rd generation catalyst [153,154]; α-pinene, β-pinene, and limonene were used to prepare terpene (meth)acrylate monomers, and the ensuing di- and multiblock copolymers were prepared by RAFT polymerization [155]; β-myrcene, an acrylic monoterpene, was submitted to anionic polymerization with D,L-limonene as a solvent, yielding polymyrcenes with narrow molecular weight distribution (Ð~1.06) [156]. The progress made in the field is indeed thanks to the exploitation of minor terpenes as platforms, and the conversion of limonene, limonene oxide, α-pinene oxide, borneol, camphor, menthol, carvone, as well as of β-pinene and other terpenes and their derivatives, into polyolefins, polycarbonates, polyesters, polyurethanes, and polyamides, was thoroughly reviewed [157].

### 4.4. Miscellaneous: Other Relevant Bio-Based Monomers 

Other aliphatic and aromatic monomers represent an equally important group of biobased molecules that may be exploited to the development of the burgeoning field of polymers from renewable resources. They often correspond to diacids (such as succinic, adipic, and itaconic acids), diols (such as 1,2-ethanediol, 1,3-propanediol, and 1,4-butanediol), diamines (as 1,4-diaminobutane and 1,5-diaminopentane), and other bi- or polyfunctional monomers, as in the case of citric acid, levulinic acid, vanillic acid and vanillin, ferulic acid, lactic and malic acids, among others [5]. 

Although many contributions to the field have been published in the last decades, many research opportunities still exist and some persistent challenges are progressively being overcome. The use of succinic acid as a precursor for polycondensation polymers, for instance, is limited due to its tendency to intra-cyclization at high temperatures. A viable synthesis of succinic acid-based polyamides with Mw up to 26,000 Da was reported, based on a direct solid-state polymerization method [158]. Also, efforts are being directed to the development of economically viable catalytic processes for the large-scale production of succinic acid from biomass [159]. Optimized and more sustainable routes to produce adipic acid are also relevant, and a new synthetic route for generating 6-hydroxyhexanoic acid, adipic acid, and ε-caprolactone from 1,6-hexanediol produced from 5-hydroxymethyl furfural, was achieved by integrating biological and chemical catalysis [160]. 

The production of itaconic acid has witnessed important progress, that started from the chemical approach, i.e., the pyrolysis of citric acid to itaconic anhydride, followed by the hydrolysis of the anhydride, and reached a situation in which microorganisms may be specifically engineered to achieve its optimal biotechnological production [161]. The polymerization of itaconic acid, in turn, was considered of limited interest up to a few years ago using conventional polyaddition initiators, but the scenario was recently changed by the possibility of using controlled radical polymerization techniques, notably atom transfer radical polymerization (ATRP) and reversible addition and fragmentation chain transfer radical polymerization (RAFT). The topic was discussed in a very interesting and complete monograph that tackled the opportunities of preparing itaconic acid-based polymers with controlled molar mass distributions and architectures [162]. 

The biological production of diamines, such as 1,3-diaminopropane, 1,4-butanediamine, 1,5-pentanediamine, 1,6-diaminohexane, 1,8-diaminooctane, 1,10-diaminodecane, and 1,12-diaminododecane, as well as other aromatic diamines, was recently reviewed [163], paving the way for a wide family of sustainable polyurethanes and polyamides. This was the case, for instance, of the polymerization of 1,4-butanediamine with different mixtures of glutaric and azelaic acids [164].

**Figure 9:**
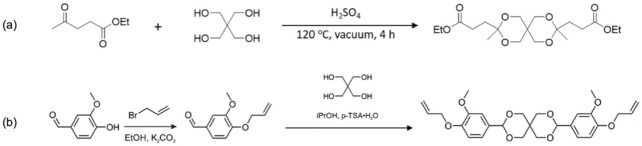
(**a**) Ketalization of levulinic acid and pentaerythritol for the synthesis of a cyclic polycondensation monomer [168]. (**b**) Synthesis of a bio-based allyl ether monomer from vanillin [167].

In recent years, the indirect exploitation of levulinic acid to the design of original polymers has gained attention. The catalytic conversion of levulinic acid into 2-hydroxy-2-methylsuccinic acid (citramalic acid) was also reported [165], which is a high-value chemical that can replace succinic acid in polybutylene succinate, enhancing its properties. The synthesis of a cyclic diester via the ketalization of levulinic acid with pentaerythritol was described (Figure 9a), and the obtained monomer was polymerized by transesterification with 1,4-butanediol, 1,6-hexanediol, neopentyl glycol, and 1,4-cyclohexanedimethanol, yielding fully amorphous polyesters with glass transition temperatures ranging from 12 to 49 °C and thermal stability up to 300 °C [166]. An analogous approach took advantage of the aromatic character of vanillin to synthesize the allyl ether monomer 3,9-bis(4-(allyloxy)-3-methoxyphenyl)-2,4,8,10-tetraoxaspiro [5.5]undecane (Figure 9b), which was mixed with thiols with different functionalities and polymerized through thiol-ene *click* photopolymerization to form the corresponding networks with tunable properties [167].

In a similar vein, ferulic acid was used as a platform to synthesize an AB-type polycondensation monomer, ferulic acid methyl (*E*)-3-(4-(2-hydroxyethoxy)-3-methoxyphenyl)acrylate, which was then polymerized with dimethyl terephthalate or dimethyl succinate, respectively, and aliphatic diols [169]. Another interesting approach considered the incorporation of the ferulic acid-derived bis-O-dihydroferuloyl-1,4-butanediol into rigid and brittle commercial-grade polylactic acid (PLA), yielding a non-covalently crosslinked elastomeric material exhibiting an elongation at break of 434%, a value that is way higher than of pristine PLA [168].

Malic acid is a natural hydroxy acid that can be found in many natural resources (such as grapes and apples) but has so far been mainly produced by chemical synthesis from fossil resources as racemic d,l-malic acid. However, the biotechnological production of malic acid has been proved viable, thanks to the ability of some fungal species of *Aspergillus*, *Ustilago*, and *Aureobasidium*, to synthesize L-malic acid from different carbon sources [170]. Therefore, the exploitation of malic acid as a building block for polymer synthesis is sought to gain increasing attention in the field of polymers from renewable resources. This was the case of a recent contribution of Yang and coworkers [171], who synthesized polyols from 1,6-hexanediol, malic acid, and citric acid, and then prepared water-blown rigid polyurethane foams with polyisocyanates by a catalyst-free method. Malic acid was also considered for the synthesis of oligoesters via reaction with ε-caprolactone and enzymatic catalysis [170].

## 5. Overview on Biodegradable Bio-Based Polymers

Biodegradation, viz., the degradation through the enzymatic action of microorganisms, of chemicals and materials once disposed into the environment, has been extensively studied and implemented in the context of polymer science for decades because of the uncontrolled accumulation of discarded materials in the land, seas, and rivers. Furthermore, the increasing effort of the industrial sector to shift towards processes and products that are less dependent on the fossil platform, mostly driven by the need of reducing carbon footprints to address the goals of the Paris Agreement, has boosted this initiative, which lights a warning sign on polymers from renewable resources and the frequent misconception concerning biodegradability.

Polysaccharides, animal- and plant-based proteins, polyesters (such as poly(lactic acid)—PLA, poly(hydroxyalkanoates)—PHAs, and poly(caprolactone)—PCL), among many other bio- and fossil-based polymers, are potentially biodegradable materials that can be exploited in the most diverse domains, such as in biomedical applications, agricultural devices and, in more general terms, in search of materials which degrade in a natural environment. However, there is no one-to-one correlation between the origin of a given polymer and its aptitude to biodegrade. In fact, Plastics Europe define bio-based plastics as (i) conventional non-biodegradable polymers, whose monomers have been produced from biomass, such as polyethylene made from sugar cane derived ethylene or polyamide made from castor oil, or (ii) special biodegradable polymers, which can decompose under the action of microorganisms [172]. Many polymers from renewable resources do indeed exhibit this property, e.g., poly(lactic acid) or starch, but others do not, e.g., natural rubber and furan polymers [6].

It is also important to mention the unfortunately frequent erroneous labeling of a polymer as “biodegradable”, when not accompanied by the precise environmental conditions. While microbial PHAs (especially poly(3-hydroxybutyrate)), for instance, show biodegradable behavior in most aerobic and anaerobic environments defined by ASTM standards, such as soil and marine environments [173], PLA is known to be recalcitrant in aquatic media, which can be attributed to different degradation mechanisms. While PHAs artifacts are attacked on the surface by enzymes (e.g., PHB depolymerases, lipases), PLA is initially degraded by a hydrolytic mechanism that does not involve enzymes and is strongly temperature dependent [174].

Although there is no intent to deepen in the mechanisms of polymer biodegradation here, since recent and complete monographs are available [175,176,177,178,179], the message aims at warning the reader that biodegradation is a complex process and must not be used for industrial and academic marketing purposes. Biodegradability is strongly dependent on the specific molecular structure of polymers, as well as on the additives used (not necessarily harmless to microorganisms) and on macrostructural characteristics, not to mention the test conditions (laboratory or full-scale) and the parameters to be accessed (weight loss, changes in thermal and mechanical properties, mineralization rate, among others) [175], which emphasizes that no deduction should be accepted without concrete data.

## 6. Conclusions

The last boom of initiatives envisaging the development of new polymers from renewable resources was initiated at the beginning of the new millennium, motivated by a sum of environmental and economic aspects. The former is related to the actual global goal of establishing general environmental benign processes, and the latter to a persistent concern with the fluctuation of the prices of petroleum, which have mainly affected emerging economies. In the present moment, one can state that renewable resources are the fundamental sources of polymeric materials for the XXI century.

During the last couple of decades, the important efforts devoted to the field allowed to reach an arguably common knowledge that the renewable origin of materials, alone, is not enough to achieve the environmental audacious goals established on the Paris agreement and at the more recent COP26. The renewable resource must be chosen wisely, on the one hand, to avoid competition with the food industry, and on the other hand, not to stimulate any kind of deforestation. Moreover, the renewable origin must be ideally associated with green overall processes, whose carbon footprint should be well monitored. In addition, it is important to consider the environmental impacts of the post-use polymer industry, and the concept of the circular economy also started becoming relevant in this field several years ago. The scenario is, therefore, very broad, and demands actions from all stakeholders—research and development, industries, governments, and final consumers.

Herein, we aimed to tackle the promising research initiatives related to ongoing investigations and potential applications dedicated to the production of original materials from animal- and plant-based resources. We briefly presented the most recent and promising contributions, supported by a notable number of review articles recently published. A careful reading of the text suggests that viable large-scale production of polymers from renewable resources is still limited by the cheap well-consolidated fossil-based analogous processes, but the scenario is expected to change in the near future, following the fluctuating tendencies in petroleum prices, as well as the mandatory actions imposed to mitigate the climate change.

## Data Availability

The study did not report any new data.
